# Tubercle-bacilli in the Feces

**Published:** 1897-01

**Authors:** 


### ABSTRACTS FROM FOREIGN JOURNALS.

UNDER THE DIRECTION OF

J. PRESTON HOSKINS, Princeton, N. J.

---

Tubercle-bacilli in the Feces. The *Bulletin Medicale* recently published a report of a series of experiments conducted for the purpose of determining whether these bacilli are to be found alive in the excreta of cattle. A young bullock was fed a meal consisting of bread and a portion of a tuberculous lung. During the three days following portions of fecal matter were collected and investigated, both by the injection of animals and microscopical examination. Bacilli were constantly found in the feces, and out of fifteen rabbits inoculated twelve became tuberculous, showing the fecal matter of tuberculous cattle to be as infectious in character as the sputum of persons suffering from this disease.—*Modern Medicine and Bacteriological Review.*

